# Diagnostic and Therapeutic Challenges of Symptomatic Meckel’s Diverticulum in a 61-Year-Old Man With Comorbid Rectal Adenocarcinoma

**DOI:** 10.7759/cureus.109353

**Published:** 2026-05-21

**Authors:** Ezekiel Kapusciak, Shiva Morisetty, Emilee Tennen, Kumar Shah

**Affiliations:** 1 Radiology, HCA East Florida Aventura Hospital, Aventura, USA; 2 Radiology, HCA East Florida Westside Hospital, Davie, USA; 3 Medicine, Nova Southeastern University Dr. Kiran C. Patel College of Allopathic Medicine, Fort Lauderdale, USA

**Keywords:** intermittent hematochezia, meckel's diverticulum, mesenteric angiography, rectal adenocarcinoma, vitelline artery

## Abstract

Meckel’s diverticulum (MD) is the most common congenital anomaly of the gastrointestinal tract, yet it remains a diagnostic challenge in adults due to its non-specific presentation and lower prevalence compared to the pediatric population. We report the case of a 61-year-old man with a history of rectosigmoid adenocarcinoma who presented with recurrent, life-threatening hematochezia from a diverting loop ileostomy. After inconclusive endoscopic and computed tomography (CT) evaluations, mesenteric angiography identified an aberrant omphalomesenteric (vitelline) artery supplying an MD. Following coil embolization for acute hemostasis and subsequent nuclear medicine confirmation, the patient underwent successful surgical resection. This case underscores the importance of maintaining a high index of suspicion for MD in adults with obscure gastrointestinal bleeding.

## Introduction

Meckel’s diverticulum (MD) results from the incomplete obliteration of the vitelline (omphalomesenteric) duct during the fifth to seventh weeks of gestation [1]. They occur in 2%-3% of patients, making them the most common congenital anomaly in the gastrointestinal (GI) tract [2]. While it follows the "rule of twos" (2% of the population, 2 inches long, 2 feet from the ileocecal valve, and 2 common ectopic tissues including gastric and pancreatic), it is primarily considered a pediatric diagnosis [3,4]. In adults, MD is often asymptomatic; however, when complications such as hemorrhage, obstruction, or diverticulitis occur, they frequently mimic other adult pathologies-such as inflammatory bowel disease or malignancy-leading to significant diagnostic delays [5,6]. The main imaging method for diagnosing MD is a technetium-99 (Tc-99m) pertechnetate scintigraphy (Meckel’s) scan [7]. However, this method has decreased sensitivity for detection in adults with GI bleeding compared to the pediatric population [8]. One common reason for lower sensitivity in adults is the decreased prevalence of ectopic gastric tissues. The ability to identify a persistent vitelline artery, strongly suggestive of MD, on mesenteric angiography can reduce delays and improve patient outcomes.

## Case presentation

Patient history and clinical presentation

A 61-year-old man with a history of hypertension, major depressive disorder, and rectosigmoid adenocarcinoma presented to the Emergency Department with acute, massive hematochezia. His surgical history was significant for a robotic-assisted lower anterior resection (LAR) with colo-anal anastomosis and a diverting loop ileostomy performed three months prior. He was currently undergoing adjuvant chemotherapy.

The patient reported four episodes of voluminous bloody output from his ileostomy. A similar, less severe presentation one month prior had been managed conservatively. On physical examination, the patient appeared weak but hemodynamically stable initially, though he soon experienced a drop in hemoglobin to a nadir of 7.0 g/dL, approximately three months after the LAR surgery, thus ruling out immediate post-operative complications. Massive transfusion protocol (MTP) was activated, and he received four units of packed RBCs (pRBCs), two units of plasma, and one unit of platelets.

Diagnostic workup

Initial computed tomography (CT) angiography (CTA) of the abdomen and pelvis (Figure 1) was unrevealing for active extravasation but noted a non-specific fluid collection around a single loop of bowel in the pelvis, later correlated to the location of MD.

**Figure 1:**
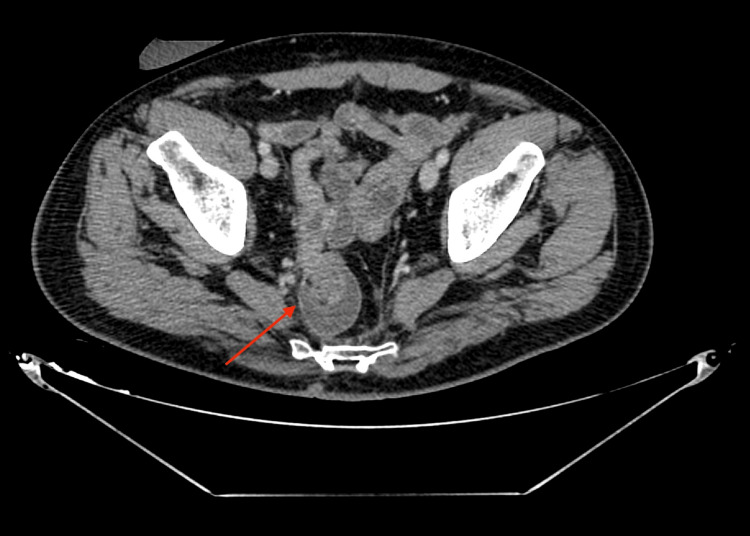
Computed tomography (CT) abdomen and pelvis with IV contrast CT abdomen and pelvis with IV contrast demonstrating a loop of bowel in the deep pelvis with a surrounding fluid attenuating collection (red arrow) of unclear significance. This fluid collection was later found to correspond with Meckel’s diverticulum (MD). On subsequent CT following diverticulum resection, the fluid collection was no longer visualized.

Additionally, a filling defect was identified in the left femoral vein, later confirmed as a deep vein thrombosis (DVT) by ultrasound. Given the active bleeding, anticoagulation was contraindicated, and an inferior vena cava (IVC) filter was planned.

The gastroenterology team performed an emergent esophagogastroduodenoscopy (EGD) and ileoscopy through the ileostomy. The distal loop (toward the colon) of the ileostomy showed arteriovenous malformation (AVM)-like formations in the region of the colon, and the proximal loop revealed a red clot with adjacent oozing. Because of poor visualization and the inability to localize a definitive source, an endoscopic clip was placed in the suspicious region for future radiological guidance.

Intervention and post-procedural imaging

The patient was transferred to Interventional Radiology (IR) for mesenteric angiography. Selective catheterization of the ileocolic artery and superior mesenteric artery (SMA) demonstrated an abnormal, tortuous, and prominent artery arising from a distal ileal branch (Figure 2) in the region of the previously placed endoscopic clip. This vessel extended beyond the vasa recta arcade and supplied a focal vascular blush (hyperemia) (Figure 3) in the right lower quadrant, associated with a dilated draining vein. These findings were highly suspicious of a persistent omphalomesenteric (vitelline) artery, suggesting an MD.

**Figure 2:**
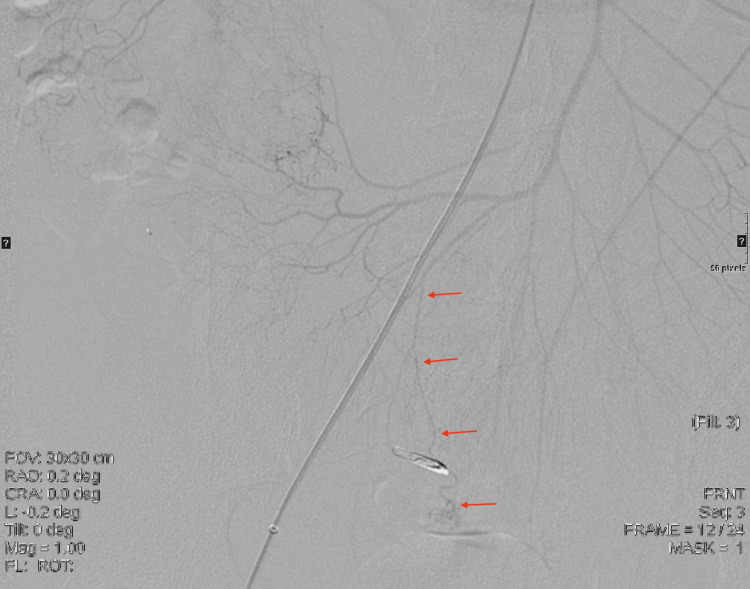
Superior mesenteric artery angiogram Superior mesenteric artery angiogram demonstrating a single tortuous aberrant ileal branch extending beyond the vasa recta arcade (red arrows). This finding is suspicious of a persistent omphalomesenteric (vitelline) artery.

**Figure 3:**
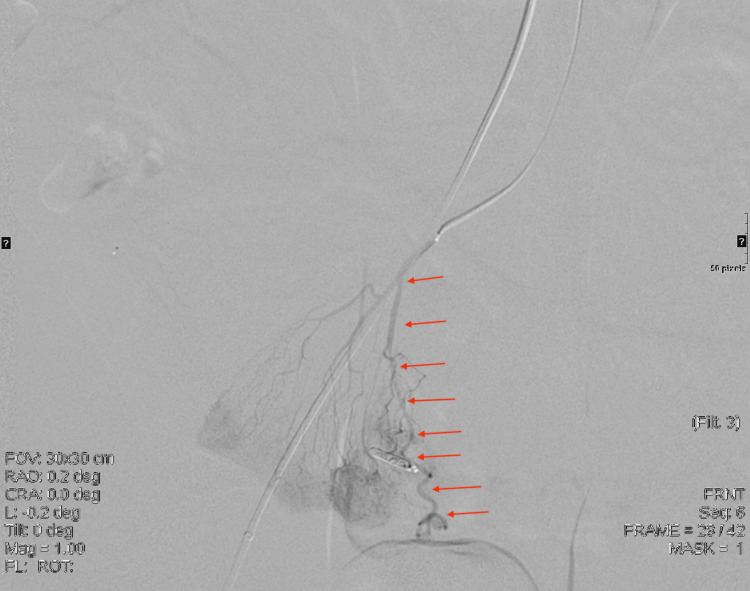
Sub-selective persistent omphalomesenteric (vitelline) artery angiogram Sub-selective angiography of the persistent omphalomesenteric (vitelline) artery (red arrows) extending beyond the vasa recta arcade supplying the suspected Meckel's diverticulum.

To achieve hemostasis, selective coil embolization of the most distal aspect of the aberrant vessel was performed with two 3 mm x 8 cm detachable coils. Distal embolization was performed to avoid potential bowel ischemia, more commonly associated with proximal embolizations. Post-embolization angiography confirmed successful occlusion. Additionally, at the time of angiography, an IVC filter was placed to address the left femoral vein DVT. Anticoagulation was held during hospital admission due to concurrent GI bleed. Approximately one day after the procedure, a Tc-99m pertechnetate scan (Meckel’s scan) was performed, which demonstrated a single focus of increased radiotracer uptake in the pelvis (Figure 4), concordant with the prior angiographic findings. Single vessel distal coil embolization reduces blood flow to a region but does not prevent all blood flow from collateral vessels; thus, the radiotracer uptake was not inhibited.

**Figure 4:**
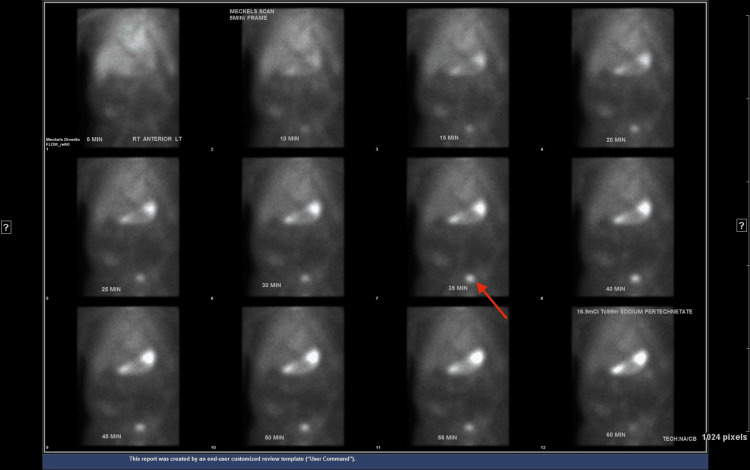
Nuclear medicine (NM) technetium-99 (Tc-99m) scan (Meckel’s scan) NM Tc-99m scan (Meckel’s scan) demonstrates a single focus of increased radiotracer uptake in the pelvis (red arrow), consistent with Meckel’s diverticulum and consistent with prior imaging location.

Management and outcomes

With the diagnosis confirmed and the patient stabilized, he was taken for surgical resection (Figure 5).

**Figure 5:**
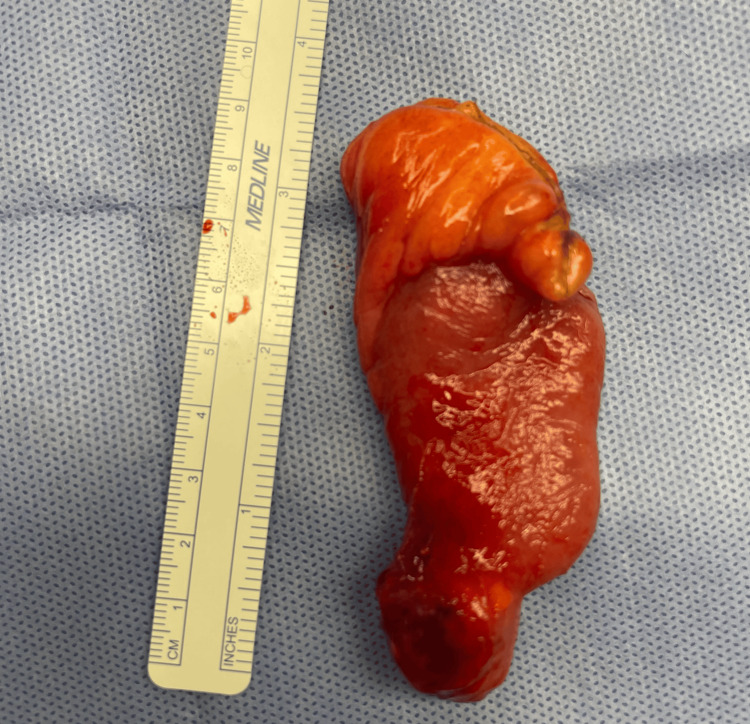
Gross specimen immediately post-surgical resection Short segment loop of small bowel with a darker red protuberance arising from the antimesenteric portion of the bowel, representing Meckel's diverticulum.

Final pathology confirmed the presence of an MD. An excerpt from the final surgical pathology report is as follows: "A segment of small bowel measuring 7 x 5 x 3 cm in dimensions. Two staple lines are observed. Protruding from the small bowel in the anti-mesenteric portion, is a tubular dark-red projection measuring 2 x 2 x 2 cm in dimension, consistent with diverticulum... The bowel is opened and the projection appears to communicate directly with the bowel lumen... The diverticulum is composed of the usual intestinal layers. The diverticular mucosa is almost completely replaced by granulation tissue and hemorrhage, with only rare foci of distal ileum type mucosa present." The patient’s postoperative course was uncomplicated, with no further episodes of GI bleeding.

## Discussion

MD is often clinically silent in adults, frequently overlooked in favor of more common pathologies such as inflammatory bowel disease, diverticulitis, or, as seen in this patient, malignancy. While the Tc-99m pertechnetate scan is the gold standard in pediatrics (with sensitivity exceeding 85%), its efficacy is considerably lower in adults. This is largely due to a lower prevalence of ectopic gastric mucosa [9] and the rapid washout of the radiotracer in cases of brisk hemorrhage. When non-invasive imaging like CT and endoscopy prove inconclusive, mesenteric angiography has the potential to be both diagnostic and therapeutic.

Mesenteric angiography serves a dual purpose: it is both a high-resolution diagnostic tool and a therapeutic intervention. In this case, the identification of an aberrant omphalomesenteric (vitelline) artery led to diagnosis, treatment of hemorrhage, and definitive therapy. The vitelline artery is typically a long, relatively non-branching vessel originating from the SMA that terminates toward the umbilicus and may be associated with hyperemia or, in certain circumstances, contrast extravasation. Its presence is strongly suggestive of MD [10].

The therapeutic versatility of angiography is best demonstrated in the management of acute GI bleeding. Embolization provides a bridge to definitive therapy, particularly in patients who are poor surgical candidates. While surgical resection of MD remains the definitive treatment, a patient in hemorrhagic shock is often a poor candidate for immediate resection.

Targeted embolization can provide immediate hemodynamic stabilization. By selectively occluding the feeding vitelline artery, an emergent, "blind" surgical exploration can be converted into a semi-elective, targeted procedure. This transition significantly reduces perioperative morbidity and allows for better surgical planning.

This case underscores that IR is not merely a "last resort" but a primary diagnostic pillar for obscure GI bleeding (OGIB). The ability to visualize the unique vascular signature of an MD allows for a definitive diagnosis when traditional imaging fails.

Furthermore, the integration of cone-beam CT (CBCT) during angiography can provide 3D anatomical localization of the diverticulum, further guiding the surgical team. As clinical practice moves toward more minimally invasive paradigms, the interventionalist’s ability to diagnose the vessel and stop the bleed is indispensable in the management of complex adult MD.

## Conclusions

Symptomatic MD should remain in the differential diagnosis for OGIB regardless of patient age. Mesenteric angiography has the potential to be a powerful tool in cases when the diagnosis is unclear and there is a need for therapeutic management of GI bleeding. Recognition of a persistent vitelline artery on angiography is an important skill for any interventionalist to aid in the diagnosis of MD. A multimodal approach, combining high-resolution CT, angiography, and nuclear medicine, as well as a multidisciplinary team consisting of gastroenterology, surgery, and IR, is often necessary to reach a definitive diagnosis.
